# The Movement of Research from the Laboratory to the Living Room: a Case Study of Public Engagement with Cognitive Science

**DOI:** 10.1007/s12152-016-9259-6

**Published:** 2016-04-28

**Authors:** Tineke Broer, Martyn Pickersgill, Ian J. Deary

**Affiliations:** Usher Institute for Population Health Sciences and Informatics, Edinburgh Medical School, University of Edinburgh, Teviot Place, Edinburgh, EH8 9AG UK; Centre for Cognitive Ageing and Cognitive Epidemiology, University of Edinburgh, Edinburgh, UK

**Keywords:** Science communication, Media coverage, Public engagement, Qualitative research

## Abstract

Media reporting of science has consequences for public debates on the ethics of research. Accordingly, it is crucial to understand how the sciences of the brain and the mind are covered in the media, and how coverage is received and negotiated. The authors report here their sociological findings from a case study of media coverage and associated reader comments of an article (‘Does bilingualism influence cognitive aging?’) from Annals of Neurology. The media attention attracted by the article was high for cognitive science; further, as associates/members of the Centre where it was produced, the authors of the research reported here had rare insight into how the scientists responsible for the Annals of Neurology article interacted with the media. The data corpus included 37 news items and 228 readers’ comments, examined via qualitative thematic analysis. Media coverage of the article was largely accurate, without merely copying the press release. Analysis of reader comments showed these to be an important resource for considering issues of import to neuroethics scholars, as well as to scientists themselves (including how science communication shapes and is shaped by ethical, epistemic, and popular discourse). In particular, the findings demonstrate how personal experiences were vital in shaping readers’ accounts of their (dis)agreements with the scientific article. Furthermore, the data show how scientific research can catalyse political discussions in ways likely unanticipated by scientists. The analysis indicates the importance of dialogue between journalists, laboratory scientists and social scientists in order to support the communication of the messages researchers intend.

## Introduction

Although the notion that public education about science will necessarily lead to acceptance of (controversial) areas of research has shown to be problematic [1–3], less goal-oriented agendas for public communication and engagement can, do and should continue to flourish [2, 4–9]. The print media has long been a key avenue for science communication [3, 8]. Yet, as well as communicating scientific findings, the job of a journalist is to attract a wide readership. Consequently, the presentation of research to fit a particular story can lead to scientists being - sometimes justifiably [10] - concerned that their research has been (or will be) represented in ways with which they are uncomfortable [6]. For their part, many journalists have experienced frustrating encounters with researchers who are resistant to placing their work in a wider context, or who struggle to articulate it in ways that will be comprehensible to non-specialists [3, 6].

Blogs, discussion forums, and the like represent newer forms of science communication, presenting various kinds of opportunities for public engagement with science [11]. It has been suggested, for instance, that blogs can act as “a watchdog when members of the public and experts comment on the quality of research and the interpretation of the results” [12] (p. 663). Further, comment forums attached to online newspapers can provide scientists with an opportunity to “clarify misrepresentation or misinterpretation of their research” [12] (p. 663). These sites of debate and dialogue can also serve as new sources of data for social scientists investigating public engagement with the (neuro)sciences.

Media communication of the sciences of the brain and the mind has an ethical dimension and bears scrutiny [13]. This is because the means by which scientific research is enrolled within news items, including the ways it is framed, have consequences for public debates about the ethical aspects and implications of innovation. In this paper, the authors examine how one widely-discussed article on cognitive aging in a prominent peer-reviewed journal was represented in news coverage, and discussed in readers’ comments forums on the webpages associated with those news items. Drawing on sociological methods and concepts, the authors analyse how readers of news items interpret and receive scientific research, and how they engage with and criticize both research findings and the manner of their reporting by journalists. In particular, the data presented in this paper demonstrate how media audience’s personal experiences are used to (de)legitimize the findings of scientific research, and how science communication can shape – or reinforce – ethical, political and social debates on topics that might at first seem distant from the scientific articles about which journalists report.

## Methods

### Case Study

The case study of the media analysis presented here is coverage of a recent article in *Annals of Neurology*. Titled ‘Does bilingualism influence cognitive aging?’, the article was led by Thomas Bak (University of Edinburgh) [14]. It drew upon the Lothian Birth Cohort 1936, the participants of which “took an intelligence test in 1947 at age 11 years” (p. 959). The authors of the *Annals of Neurology* article re-tested the cohort in 2008–2010 on general intelligence, memory, speed of information processing, verbal reasoning, vocabulary/reading, and verbal fluency. The participants were generally healthy older people without dementia; hence, the study investigated the effect on the normal range of cognitive ageing of bilingualism (rather than on dementia). In total, 853 of the 1091 participants of the Lothian Birth Cohort 1936 completed the bilingualism questionnaire which determined whether or not they had learned any languages other than English and the extent to which they actively used the second language. Two hundred sixty-two participants “reported having learned at least 1 language other than English to a degree allowing them to communicate” (p. 961).

Based on this sample, the authors showed that bilingualism is related to better cognitive skills in older age, including in those who learned their second language in adulthood. Bak et al. furthermore demonstrated that this relation could not be explained only by childhood intelligence (i.e., they ruled out the possibility that it was more intelligent children who tended to learn a second language and who continued to be more intelligent in older age). These findings suggest that the answer to Bak et al’s title question is ‘yes’: bilingualism indeed influences cognitive aging. In the article discussion, it was hypothesised that the observed “effects on general intelligence are likely to be related to frontal executive advantages, the best documented nonverbal cognitive feature of bilingualism” (p. 962). It was furthermore suggested that the data should be interpreted from a cognitive epidemiology perspective, “rather than clinical application to an individual”, with changes in the number of people in a population able to speak an additional language likely to have a population effect on pathology rates related to cognitive ageing (p. 962).

The Bak et al. article is the focus of the media analysis presented here for two key (among other) reasons. First, the amount of public attention the article attracted was high for this type of study.^1^ It was noted in a range of countries and different types of newspapers and websites. The media coverage itself elicited many readers’ comments on the various online news items. Second, the paper was produced as part of the University of Edinburgh’s Centre for Ageing and Cognitive Epidemiology (CCACE). The authors of this analysis are associates/employees of CCACE, enabling direct discussion with Bak himself regarding the media attention he received. The first author also attended a video shoot with Bak where he was interviewed about the *Annals of Neurology* article. This granted additional contextual insight into how his encounters with the media played out, and to what ends. Accordingly, the article by Bak and its media coverage were not selected on the basis that these were necessarily representative of the way scientific research more generally is covered. However, this coverage will nevertheless have some generalizability to other scientific studies of the brain and mind [15]. As O’Connor and Joffe argue, a “detailed investigation of one particular case can (…) be a potent means of exposing the naturalistic unfolding of the processes of science communication” [11] (p. 3).

### Data Collection and Analysis

The Lexus Nexus database (containing many news articles published around the world) was searched in order to locate coverage of the Bak et al. article. The results were cross-checked via a Google search, and via the database of the press communication officer responsible for the press release of the Bak et al. article. Mentions of the article were found in over 400 websites and newspapers. Many either reprinted an unedited press release, or reprinted the coverage as it appeared on another website. An electronic corpus of relevant English-language news items was compiled. This incudes items published both in newspapers and in recognised online news sources (e.g. *Huffington Post*). Any readers’ comments relating to the news items were also included within the corpus.

A total of 37 news items were included in the corpus. These were published in 14 different countries (Bolivia, Canada, China, Egypt, France, India, Kenya, Kyrgyzstan, Malaysia, Malta, South Korea, United Arabian Emirates, the UK, and the US). The shortest item was 97 words; the longest, 859 (median article length: 405 words). Where readers’ comments were included (in 9 sources), the lowest number of comments was 1 (*Egypt Independent*) and the highest was 103 (*The Telegraph*). The median number of comments was 4. In total, 228 comments were incorporated in the dataset. Table 1 presents an overview of all the news items, their titles, sources, date, and number of words and comments.

**Table 1:** Overview included articles

Article title	Newspaper	Date	Number of words/comments	Percentage similarity to press release^a^
Bolivia				
More benefits of bilingualism discovered	*El Diario*	June 3, 2014	587/0	7 % By-line: Hope Gillette
Canada				
Bilingualism boosts the brain at all ages	*CBC News*	June 29, 2014	594/0	3 % By-line: CBC News
China				
Study: Adult language learning can prevent atrophy of the brain	*China Daily*	August 2, 2014	480/0	26 % By-line: Lucy Kinder
Egypt				
A second language may keep the brain young	*Egypt Independent*	June 4, 2014	633/1	3 % By-line: (Reuters)
France				
Another case for bilingualism: healthy brain aging	*AFP - RELAXNEWS (English International Version)*	June 3, 2014	371/0	6 % By-line: None
India				
Speaking two languages keeps brain’s ageing at bay	*Business Standard*	June 2, 2014	370/0	11 % By-line: None
Speaking two languages keeps brain’s aging at bay	*The Times of India*	June 2, 2014	328/30	11 % By-line: None
Being bilingual can slow brain ageing	*The Financial Express*	June 2, 2014	249/0	11 % By-line: None
Kenya				
More the merrier is thinking on languages	*The Standard*	June 4, 2014	114/0	0 % By-line: None
Kyrgyzstan				
Learning second language slows brain ageing - study	*AKIpress News Agency (Kyrgyzstan)*	June 2, 2014	211/0	0 % By-line: None
Malaysia				
Another case for bilingualism: Healthy brain aging	*Malay Mail Online*	June 4, 2014	377/0	6 % By-line: None
Learn more languages for healthy brain aging	*Free Malaysia Today*	June 4, 2014	399/0	6 % By-line: (AFP Relaxnews)
Malta				
Learning another language may help the ageing brain	*The Times of Malta*	June 4, 2014	644/2	3 % By-line: Kathryn Doyle
South-Korea				
Learning a second language slows brain aging	*The Korea Times*	June 29, 2014	154/0	10 % By-line: Jeong Ji-su
United Arab Emirates				
Speaking two languages keeps brain’s ageing at bay	*Khaleej Times*	June 3, 2014	336/0	11 % By-line: None
United Kingdom				
Speaking two languages ‘slows brain ageing’; Researchers from the University of Edinburgh found people who spoke two or more languages had significantly better cognitive skills later in life	*The Independent*	June 3, 2014	287/19	37 % By-line: Heather Saul
Being bilingual ‘keeps brain sharp’	*The Daily Telegraph*	June 2, 2014	187/0	8 % By-line: None
Being bilingual boosts brain power	*Daily Express*	June 2, 2014	212/4	4 % By-line: Dan Townend
Being bilingual may keep brain sharp in old age: Learning extra languages can help prevent onset of dementia, study claims	*Daily Mail*	June 2, 2014	419/64	10 % By-line: Ben Spencer
Mind your language, it’ll help in old age	*Metro (UK)*	June 2, 2014	220/0	7 % By-line: None
Being bilingual boosts brains	*Scottish Express*	June 2, 2014	201/0	4 % By-line: Dan Townend
Bingo? I’ll take lingo	*The Sun (England)*	June 2, 2014	97/0	0 % By-line: None
Learning a second language in adulthood can slow brain ageing; Speaking two languages benefits the ageing brain, according to new research, and it can be just as beneficial to learn one later in life as in childhood	*The Telegraph*	June 2, 2014	463/103	25 % By-line: Lucy Kinder
Being bilingual can keep mind sharp	*The Times*	June 2, 2014	343/0	11 % By-line: Katie Gibbons
Learning second language ‘slows brain ageing’	*BBC Health Online*	June 2, 2014	462/0	11 % By-line: None
Speaking Two Languages May Slow Brain Aging	*Huffington Post (UK)*	June 2, 2014	474/0	8 % By-line: Shelley Emling
A second language may keep the brain young	*Yahoo News*	June 2, 2014	644/2	3 % By-line: Kathryn Doyle
United States				
Learning a second language at any age may slow the brain’s decline	*Chicago Tribune*	June 2, 2014	751/0	2 % By-line: Amina Khan
Learning a second language at any age may slow the brain’s decline	*Daily Press*	June 2, 2014	751/0	2 % By-line: Amina Khan
Learning a second language at any age may slow the brain’s decline	*Las Vegas Sun*	June 3, 2014	652/0	3 % By-line: Amina Khan
Learning a second language at any age may slow the brain’s decline	*LA Times*	June 3, 2014	859/0	2 % By-line: Amina Kahn
Learning a second language at any age may slow the brain’s decline	*The Baltimore Sun*	June 2, 2014	784/0	2 % By-line: Amina Kahn
Learning a second language at any age may slow the brain’s decline	*The Virginia Gazette*	June 2, 2014	784/0	2 % By-line: Amina Kahn
A second language may help sustain the brain	*The Washington Post*	June 9, 2014	586/0	3 % By-line: Kathryn Doyle
Speaking Second Language Slows Mental Aging	*Latin Post*	June 2, 2014	369/3	51 % By-line: Erik Derr
No particular country				
Speaking 2 languages benefits the aging brain	*EurekAlert!*	June 2, 2014	510/0	16 % By-line:
New Study: Being Bilingual Slows Down Brain Ageing	*International Business Times*	June 3, 2014	405/0	8 % By-line: Roshni Mahesh
Total:			16679/228	

^a^To calculate this, we used the programme COP Doc and compared each media article with the original press release, with the programme giving us the percentage of similarity for each individual media article.

The news items and associated readers’ comments were subjected to a qualitative thematic analysis [16], used “for identifying, analysing and reporting patterns (themes) within data” [17] (p. 79). As is common in media analyses, the analytic approach was inductive: the analysis employed relevant sociological theory in order to isolate and understand key themes from the data (and the texts were not approached with particular hypotheses to be tested) [18] (p. 2479). The analysis began with detailed, line-by-line coding of the news items and reader comments, and from these codes groupings were then made (i.e. higher-order discursive themes). In particular, the analysis focused on similarities and differences in opinions expressed, seeking to capture the range of media coverage and commenting practices. Negative cases were also sought; i.e., cases that were different from the general tendency in the data or “aspects from the developing analysis that were initially less obvious” [19] (p. 18). This is a common strategy in qualitative analysis to ensure validity [20].

Finally, in line with suggestions that news coverage of scientific articles is often (too) dependent on press releases [21, 22], the extent to which the news items used the exact words of the Bak et al. press release was examined. This was achieved through the programme COP Doc, which compares documents to each other and calculates the correlation between them (in a similar way to how the programme TurnItIn can help indicate plagiarism in documents such as student essays). In the last column of Table 1, the percentage for each news item as compared to the press release is shown.

In the next sections, the authors first address the media coverage of the Bak et al article, after which the key themes in the readers’ comments are analysed. Regarding the latter, quotes are reproduced as they appeared, without correcting for spelling or grammar. In total, the Results section consists of 4 sub-sections, followed by the Discussion.

## Results

### Media Coverage

Many scientists are concerned that journalists will not accurately report their research [21]. However, the authors of this study found that news items concerning the ‘Does bilingualism influence cognitive aging?’ article were scientifically accurate.^2^ None of the news items included in the sample were exact copies of the press release.^3^ Using the COP Doc software, it was found that the highest percentage similarity between the news items in the sample and the Bak et al press release was a correlation of 51 %. This was for an item published in the *Latin Post* (USA). In general, items published in UK-media were marginally closer to the press release than those from non-English speaking countries. Elsewhere, newspapers often repeated items written by other papers; the reporter Amina Kahn, for example, wrote an article for the *Los Angeles Times* that was then reprinted word-for-word by some other US news outlets (e.g. *Chicago Tribune, Las Vegas Sun*). One article, written by Kathryn Doyle of *Reuters Health*, was reprinted in different countries, such as the UK-based *Yahoo News*, *The Washington Post* in the US, the *Egypt Independent*, and *The Times of Malta*. The extent to which a news article was similar to the press release did not appear to relate to whether or not a by-line was included. The aforementioned *Latin Post* article, for instance, included a by-line and had a correlation of 51 % with the press release, while other articles with low correlation had no by-line (such as an item in *The Sun* (England) which had a correlation of 0 %).

As an exception to the generally accurate reporting, at least one of the news items conflated cognitive skill as measured in healthy older people with dementia. In doing so, journalists could claim that speaking more languages helped to stave off dementia. The *El Diario*, a partly English newspaper serving Bolivia, wrote: “According to research published in the *Annals of Neurology*, bilingualism – even when the second language is learned in adulthood– may slow down age-related cognitive decline and dementia.” (*El Diario*, 03/06/2014)

The headlines of the media news items were largely accurate. However, the *Daily Mail* (England) was an exception; it introduced the topic of dementia in its title: ‘Being bilingual may keep brain sharp in old age: Learning extra languages can help prevent onset of dementia, study claims’ (02/06/2014). Towards the end of the *Daily Mail* item it became clear that it referred to an earlier study in which Bak was involved, as well as the *Annals of Neurology* article that formed the target of the analysis presented here. The earlier Bak article had in fact demonstrated the effects of bilingualism on the onset of dementia [23]. The coverage of the *Daily Mail* therefore was not strictly incorrect as such, although arguably misleading. Since media audiences often do not tend to read an entire news item, the placing of caveats and related rhetorical devices towards the end of reports is a frequently adopted practice in media coverage of research (and in press releases) [22]. The fact that dementia was mentioned in the title of the *Daily Mail* item could suggest that, for its readers, dementia has sensationalist value in ways that the notion of cognitive skills in general does not, relating to the emergence of dementia as a cause of public concern and anxiety [24].

A minority of the news items discussed the mechanisms behind the effects of bilingualism on cognitive skills. Many of these did so by quoting Thomas Bak. This is illustrated below:

“I don’t think there’s anything magic about learning languages,” Bak said. “Both mental and physical activity throughout the life are protective, and learning language is a very good form of brain training.” Learning a second language improves certain mental functions, mostly those connected to the frontal lobe of the brain, but not all functions, Craik [who was introduced in this article as a “senior scientist at the Rotman Research Institute at the Baycrest academic health sciences center, affiliated with the University of Toronto”] said. “It does improve fluid intelligence and ‘executive functioning,’ because you have to control the two languages you know,” he said. “While you communicate in one language you’ve got to manage and control the other language.”

(*Times of Malta*, 04/06/2014)

Another trend in the news items was a tendency to reflect on the implications of Bak et al for individual cognitive health. An item in *Free Malaysia Today*, for example, was titled ‘Learn more languages for healthy brain ageing’ (04/06/2014), suggesting that the effect of bilingualism on cognitive skills worked on individual level. Similarly, *The Virginia Gazette* item titled ‘Learning a second language at any age may slow the brain’s decline’ (02/06/2014) ended with the following sentence: “Maybe it’s time to blow the dust off of that Spanish textbook or dig up that Mandarin audio CD and learn something new.” However, in the original Bak et al article, the effects on cognitive skills were described as a population rather than an individual effect.

Indeed, this was correctly noted in some of the news items (often, again, by quoting Bak). For instance, the *Reuters Health* item that appeared in the *Egypt Independent*, amongst other news sites, concluded:

“For people who are beyond school age but are still interested in learning a second language, these results should be encouraging, Bak said. He wouldn’t recommend that people worried about dementia go out and learn another language as a precaution, but for those who are interested in language there may be unexpected benefits. “I would not push it forward but if you leave the chance of many languages being spoken in the house, it could be great brain training and great fun,” he said.” (*Egypt Independent*, 02/062014)

This extract is illustrative of the largely accurate reporting on the epidemiological nature of the Bak et al article.

In sum, while worries have been frequently articulated regarding the risk of “media exaggeration” in science communications [25], the coverage of Bak et al does not confirm such concerns (notwithstanding some exceptions discussed above). Furthermore, later in this paper, it will be shown too that there was in fact a more critical ‘downplay’ of Bak et al’s findings among many readers of news items. The next three Results sub-sections all report on readers’ comments, divided into three key themes: Readers’ comments about the Bak et al study including appreciating the study or being sceptical towards it; interpreting the findings including using personal experiences to agree or disagree with Bak et al’s findings; and Politics and the status of language. The analysis presented is qualitative, situated broadly within the interpretivist tradition of the sociology of knowledge. Table 2 also details how many comments from which sources were classified among each key theme.

**Table 2:** Description of the number of comments per category and sub-category^a^

	Egypt Independent (1 comment)	The Times of India (30 comments)	The Times of Malta (2 comments)	The Independent (19 comments)	Daily Express (4 comments)	Daily Mail (64 comments)	The Telegraph (103 comments)	Yahoo News (2 comments)	Latin Post (3 comments)	Total
Readers’ comments about the study (from appreciation to scepticism)	-	16	2	4	–	7	11	1	2	43
Appreciation	–	13	2	2	–	2	5	1	–	25
Scepticism	–	3	–	2	–	5	6	–	2	18
Making sense of the findings	–	7	2	–	1	9	39	1	–	59
Hypotheses about the study	–	2	–	–	–	1	29	1	–	33
Personal experiences	–	5	2	–	1	8	10	–	–	26
Politics	–	–	–	2	2	17	11	–	–	32
Total	–	23	4	6	3	33	61	2	2	134

^a^Some comments were classed in more than one category

### Readers’ Comments About the Bak et al Study

Few overt opinions about the Bak et al article were apparent within its media coverage, which confirms the findings of other analyses of science reporting [26]. However, the comments sections of the different news sites served as a platform for sometimes heated discussions. The identity of posters remains anonymous, but the content of comments suggests that a minority were scientists – or at least individuals with some scientific literacy [8].

The majority of those who read the media coverage of Bak et al’s study appeared appreciative of his work. A reader of *The Independent*, for example, said (perhaps slightly humorously): “That's good news. I shall carry on with my Norwegian classes.” (*The Independent*, 03/06/2014) Notably, most of this appreciation related to readers agreeing with the study based on personal experiences, or because it addressed concerns they had about their aging brains.

However, a smaller number of comments were from readers who more critically engaged with either the Bak et al article specifically, or with its media coverage. Some were cautious in interpreting the study while not immediately dismissing the findings. For instance, some readers discussed the number of people that participated in the study:

“There are no results listed here, and 835 people seems awfully small number to draw a conclusion from.”

“835 is an “awfully small number”? You’ve gotta be joking. Or don’t know much about statistics. Lothian Birth Cohorts are some of the largest cohorts ever used in geriatric studies. Especially considering that these are pre-WWII generations and 1921 cohort was old enough to be conscripted in 1939.”

(*Daily Mail*, 02/06/2014)

The above discussion illustrates one criticism of the Bak et al article (i.e. its sample size), as well as how other readers defended it.

Further, one reader commented on the issue of causality, dismissing the conclusions around this proffered by Bak et al. This reader ended their comment with the words: “Additionally, despite the conclusions given, there does not seem to be a basis for assuming causative effect e.g. people who are more intelligent and who read more might just be more likely to learn a second language?” (*The Independent*, 03/06/2014)

Others expressed less epistemologically-orientated critique, drawing instead upon assumptions about the ontology of brains. Such comments demonstrated the extent to which everyday understandings of the brain are used to make sense of findings [27, 28]:

“Of course it’s nice to have a second language but I don’t beleive this science twaddle for one second. The human brain can only contain a finite amount of information and as English speakers we are fortunate not to need a secondary language. That space is much better utilised for science, history and our rich culture.”

And a reply:

“And what space are you referring to? The brain? We cannot even fathom all the brain can store, ooops I forgot, you only speak one language!!”

(*Daily Mail*, 02/06/2014)

Comments about the Bak et al. study and about the media reporting on it were therefore diverse, ranging from (largely) appreciation to (occasionally) scepticism. Sarcasm was in some cases employed as one means of disagreeing with the findings; more frequently, it was also used between readers when commenting on each other’s posts [8]. Whereas some readers seemed to have good scientific literacy, others employed everyday conceptualizations of the brain when interpreting and discussing the Bak et al. study (of course, these different ways of understanding and engaging with neuroscience and cognitive research are not mutually exclusive). In the next section, this point is expanded upon in more detail.

### Media Audiences’ Sense-Making of the Bak et al Study

Readers of the media coverage of Bak et al frequently hypothesised about *how* bilingualism influences cognitive aging. Bak et al made the following hypothesis: “If bilinguals automatically and unconsciously activate both languages, they constantly need to select, monitor, and suppress linguistic information, stimulating frontal executive functions” [14] (p. 962; footnotes deleted). Readers of news items sometimes coined a similar hypothesis, and stimulating the brain in general was deemed important for maintaining cognitive health. As a reader of *The Times of India* stated: “The whole idea is to not let one’s brain rust. Keep it busy.” (*The Times of India*, 02/06/2014) Underscoring the range of ideas about the brain that feature within public discourse [29–31], some readers had their own advice about how to keep cognitively fit: “Whether its mastery of a language or cross words or advanced reasoning-it all keeps the brain trying to remain active.” (*Daily Mail*, 02/06/2014)

A similar discussion took place in the comment pages of *The Telegraph* (UK) (02/06/2014), in which a series of comments focused on comparing math skills with language skills. The following excerpt from this discussion illustrates the arguments posed for or against preferring maths above language:

“I find learning languages relatively easy and mathematical problems challenging. I suspect that maths problems would therefore be better for me.”

“There are several variables in the equation - not just the nature of the subject [language v mathematics].”

And, a few comments below the above remarks:

“I am afraid that your definition of language is way too simple and does not capture the real complexity of language. Memorisation is important, but solving mathematical problems does not involve developing your perception of new sounds never found in your language, as well the development of new articulatory habits while producing these sounds. Furthermore, you are forgetting about the new opportunities for social interaction language offers. To truly learn a new language, you have to engage in meaningful conversations with other nonnative or native speakers, making use of social strategies to achieve the purposes of the conversation. Solving mathematical problems may be beneficial, but it doesn't give you this mental workout (training many areas of the brain, those related to the perception and production of sounds, areas dealing with emotions and social interaction, as well as training memory and the learning of procedural knowledge while learning grammar rules). (…)”

Thus, learning a new language was understood to be one way of giving oneself a “mental workout”, aligning with the hypothesis of Bak et al – but also expanding it, since the above reader considered that learning a language also involved the use of “social strategies” in order to produce meaningful conversation rather than merely communication

Others expressed more novel hypotheses to explain the results of Bak et al. For instance, another reader of *The Times of India* wrote:

“Yes, quite possibly [Bak et al’s findings] can be true. As a person who speaks more than one language is tend to be more happy physiologically, which in turn helps the body physically. On the other hand person who speaks just 1 language isolates himself from others who speak diff language which internally makes him sad and which could be an bad effect on the body.” (*The Times of India*, 02/06/2014)

Personal experiences appeared to play a considerable role in how readers adjudicated the veracity of Bak et al’s findings, with a great many readers’ comments structured by anecdote, as in the following comment: “Agreed… my mother is 86, she does word games and other puzzles every day and her mind is as sharp as ever.” (*Daily Mail*, 02/06/2014) Likewise, personal experiences were leveraged to dismiss the study findings, as in the following comment from the *Daily Mail* (02/06/2014): “What a load of nonsense. My Dad spoke 8 languages and Alzheimers.”

The fact that people have experience with knowing different languages also gave rise to them wondering whether their particular situation fitted in the definition of ‘bilingualism’ used in the study, or whether their situation would have beneficial effects on cognitive skills. In particular, anecdotes served to underscore the hopes that some people felt regarding studies like that of Bak et al for retaining one’s cognitive skills: “I’m fluent in English, French and Mandarin. I guess I’ll be fine. Learning a new language is a lot of fun and I encourage everyone to at least give it a try.” (*Daily Mail*, 02/06/2014)

A minority of readers explicitly orientated their comments to concerns over cognitive decline, and hence to the potential ‘therapeutic promise’ [32] that some readers could ascribe to (the coverage of) the Bak et al paper: “As an old man with all my marbles (for the time being), I should dearly like to know whether being multi-lingual has any effect on the incidence of or vulnerability to Parkinsons or Alzheimers.” (*The Independent*, 03/06/2014). Such comments underscore wider public fears about the personal and societal burdens of dementia [24].

### Politics and the Status of Language

Many readers’ comments, particularly in the *Daily Mail* (a popular, politically right-leaning UK newspaper), also focused on the politics of language.^4^ Especially (apparently) native English anglophones felt the need to comment on English, or were reproached by other readers about their lack of language skills and motivation. The following discussion in the comments’ section of the *Daily Mail* (02/06/2014) is illustrative:

“I can speak the language of several countries, so I am safe from dementia!: Canada, the UK, Australia, the US….”

And:

“It’s as funny as food poisoning. I really don’t know why a lot of English expect the world to speak their language. What that attitude does do is show them up as narrow-minded, xenophobic and just plain ignorant!”

At a time where immigration is a contested political issue in the UK (and elsewhere), the Bak et al study acted as a catalyst for discussing (the language skills of) immigrants, as evidenced by the following comment on the same item in the *Daily Mail*:

“Learning the language of your country of residence would be a good start, not jabbering away and conveniently not being able to speak English unless when being told where to sign for the next handout.”

Such sentiments were apparent too in *The Telegraph* (02/06/2014), also right-leaning, as the following discussion between three (presumably different) readers demonstrates:

“Hey, (name of previous commenter)…many of the “resident aliens” at my place of work *refuse* to even *try* to learn English. Guess what will happen should an emergency arise, and these idiots need to evacuate the building?”

In reply:

“Worse yet: what would happen if THEY saw there was fire in the building. Do you think these uncomprehending, “no-speak-English” useless jag offs are going to be able to get the word out, let alone understand how to pull the fire alarm????”

And finally:

“Depends on loud they scream and how stubborn you are when someone screams “fuego!” vs. “fire!”"

In similar ways to individuals who disputed Bak et al based on their personal experiences, readers of the UK Daily Express (again, a right-leaning newspaper) who deployed the figure of ‘the immigrant’ in their comments asserted that speaking a second language did not enhance “brain power”:

“What a load of rubbish. Half the immigrant who come to the UK speak more than one language but they are not bright. linguistic skills and brain power are two different things.” (*Daily Express*, 02/06/2014)

However, the factual statements made in the media reporting of Bak et al were also politicised in ways that appeared (albeit tangentially) more positive of immigration. This included a presumably facetious comment on *The Independent* website (a left-wing UK newspaper) made about the UK Independence Party (UKIP; well-known for its negative stance towards immigration and immigrants):

““Over one million people in the UK aged 65 and over are estimated to have some degree of cognitive impairment.”

That’s self-evident: that’s the UKIP vote.” (*The Independent*, 03/06/2014)

In sum, some of the commentary on the coverage of Bak et al can be seen to link with political discourses on immigration. The mobilisation of science in this way has a long history [11, 33, 34], and its continuance is a reminder of how even carefully framed interviews and press releases can sometimes be repurposed to support ends with which scientists themselves might be uncomfortable.

## Discussion

This paper has reported on the media coverage of a recent article in cognitive epidemiology (‘Does bilingualism influence cognitive aging?’, Bak et al, 2014, *Annals of Neurology*) and the comments news readers made in response to this. The Bak et al article received considerable media attention: it was covered in major newspapers in the UK and abroad, resulting in 37 English-language items in reputable news sources. Nine of these included an online commenting forum, and a total of 228 readers’ comments were associated with news items. The analysis presented here has explored, drawing on sociological methods and concepts, how Bak et al was framed in the media, and interpreted by readers.

It was found that the reporting of Bak et al was generally accurate. This confirms Taylor et al’s [21] analysis of media coverage of an article on pancreatic cancer and processed meat [35]. Taylor and colleagues also found that many news items were direct or slightly modified copies of the study press release. The reliance on press releases for science news stories has also been noted by other authors investigating science journalism, and is often invoked as a criticism [21, 22]. Some of the more esoteric sites excluded from the sample considered here (as a consequence of their obscurity) indeed duplicated the press release. However, most of the news items included within the sample did not merely copy the press release (with the highest correlation between an article and the press release being 51 %). It is suggested that the general accuracy in the reporting might partly relate to Thomas Bak’s efforts in communicating with the media, going as far as staying in a hotel away from his family so that he would be available at all times for media requests. In this respect, the findings underscore the importance of academics working very closely with university press officers in order to ensure accurate and temperate press releases, and suggest the need for careful and sustained engagement by scientists with the media following the launch of a press release [6].

Readers’ comments on the news items surveyed were interesting in several aspects. For example, comments were made on the accuracy of the Bak et al study (and, in a few cases, on the accuracy of the media reporting). More importantly, readers’ comments illustrate how media audiences make sense of research findings. Personal experiences were especially dominant in shaping readers’ accounts of their (dis)agreements with the findings of Bak et al; deploying such experiences in commentaries can be seen as one way of establishing authority in commenting on the article [8], as well as of making sense of findings by integrating the results into existing knowledge [5, 7, 27, 36, 37]. Personal experiences were also drawn upon when investing ‘therapeutic promise’ in discovery science (e.g. speculating about the delaying of dementia or about keeping one’s cognitive health) [32]. The findings suggest that non-specialists do not always comprehend the differences between population and individual level findings, and that they (sometimes as a consequence) draw on individual experiences to cast doubt on the veracity of science.

Furthermore, the data show how scientific findings can provide a platform for heated political discussions. Societal discourse around immigration in particular provided substrates both for positions around Bak et al. to develop, and for developing further discussion on this subject. For example, the reported results of Bak et al. were sometimes used as an argument against immigration. O’Connor and Joffe have similarly shown how scientific articles can further stimulate political debates in readers’ comments [11]. In their analysis of how an article on sex differences in the brain was reported in traditional media outlets, as well as in blogs and reader comments [38], O’Connor and Joffe showed that the article was frequently related to more general themes of gender politics [11]. However, in the case study reported in this paper, the political debate that was introduced was only slightly related to the scientific article being reported and discussed. Science, then, interweaves with wider societal and ethical discourses and concerns in ways that may not be anticipated by scientists themselves. When communicating with the media, neuroscientists could afford benefit from prior and on-going discussions with social scientists about the societal themes readers might connect scientific research with. In doing so, their messages can then be framed in ways that will make them less amenable to be used by citizens to inflame already heated debates.

Finally, as science communication relies more heavily on the internet than previously [3], the case study presented here illuminates the importance of reader comments as an interesting and useful resource for studying media and audience engagement with science, and the ethical dimensions of science communication [8, 11, 12]. While the media has long been an important conduit of communication, new opportunities for responding to news items can be seen as facilitating a more interactionist form of science communication in which publics also communicate scientific notions to each other - and, perhaps, to journalists and scientists as well.

## Footnotes

1 In the experience of a press officer from our university who has been involved in keeping track of the coverage of many articles including many related to cognitive neuropsychology.

2 Indeed, Bak himself was generally pleased with the way his paper was reported.

3 Although some more esoteric websites (not included in the sample, e.g. ‘Optimist World’ or ‘Health Canal’) did merely (except for a few small changes) reprint the press release.

4 While the data for this analysis excludes non-English-language news items, it is noteworthy that Thomas Bak found that newspapers in countries in which language is explicitly a political issue, such as Spain, were more likely to request an interview from him.

## References

[1] Kahan DM, Peters E, Wittlin M, Slovic P, Larrimore Ouellette L, Braman D, Mandel G (2012). The polarizing impact of science literacy and numeracy on perceived climate change risks. Nature Climate Change.

[2] Nisbet MC, Scheufele DA (2009). What’s next for science communication? Promising directions and lingering distractions. American Journal of Botany.

[3] Weigold MF (2001). Communicating science A review of the literature. Science Communication.

[4] Abbott A. 1990. Positivism and interpretation in sociology: Lessons for sociologists from the history of stress research. Paper presented at the Sociological Forum.

[5] Cunningham-Burley S (2006). Public knowledge and public trust. Community Genetics.

[6] Illes, J., M.A. Moser, J.B. McCormick, E. Racine, S. Blakeslee, A. Caplan, E.C. Hayden, J. Ingram, T. Lohwater, P. McKnight, C. Nicholson, A. Phillips, K.D. Sauvé, E. Snell, and S. Weiss. 2010. Neurotalk: Improving the communication of neuroscience research. *Nature Reviews Neuroscience* 11(1): 61–69. 10.1038/nrn2773 PMC2818800 19953102

[7] Kerr A, Cunningham-Burley S, Tutton R (2007). Shifting subject positions experts and lay people in public dialogue. Social Studies of Science.

[8] Kouper I (2010). Science blogs and public engagement with science: Practices, challenges, and opportunities. Journal of Science Communication.

[9] Pickersgill M (2011). Research, engagement and public bioethics: Promoting socially robust science. Journal of Medical Ethics.

[10] Deary, I.J., M.C. Whiteman, and F.G. Fowkes. 1998. Medical research and the popular media. *Lancet* 351: 1726–1727. 10.1016/s0140-6736(97)10317-8 9734905

[11] O’Connor C, Joffe H (2014). Gender on the brain: A case study of science communication in the new media environment. PLoS ONE.

[12] O’Connell G, De Wilde J, Haley J, Shuler K, Schafer B, Sandercock P, Wardlaw JM (2011). The brain, the science and the media. EMBO Reports.

[13] Illes J, Bird SJ (2006). Neuroethics: A modern context for ethics in neuroscience. Trends in Neurosciences.

[14] Bak TH, Nissan JJ, Allerhand MM, Deary IJ (2014). Does bilingualism influence cognitive aging?. Annals of Neurology.

[15] Polit DF, Beck CT (2010). Generalization in quantitative and qualitative research: Myths and strategies. International Journal of Nursing Studies.

[16] Vaismoradi M, Turunen H, Bondas T (2013). Content analysis and thematic analysis: Implications for conducting a qualitative descriptive study. Nursing & Health Sciences.

[17] Braun V, Clarke V (2006). Using thematic analysis in psychology. Qualitative Research in Psychology.

[18] Gough B (2006). Try to be healthy, but don’t forgo your masculinity: Deconstructing men’s health discourse in the media. Social Science & Medicine.

[19] Morse JM, Barrett M, Mayan M, Olson K, Spiers J (2008). Verification strategies for establishing reliability and validity in qualitative research. International Journal of Qualitative Methods.

[20] Mays N, Pope C (2000). Assessing quality in qualitative research. British Medical Journal International Edition.

[21] Taylor, J.W., M. Long, E. Ashley, A. Denning, B. Gout, K. Hansen, T. Huws, L. Jennings, S. Quinn, P. Sarkies, A. Wojtowicz, and P. M. Newton. 2015. When medical news comes from press releases—A case study of pancreatic cancer and processed meat. *PLoS ONE* 10(6), e0127848. doi:10.1371/journal.pone.0127848. 10.1371/journal.pone.0127848 PMC4471125 26083640

[22] Samuel G, Williams C, Gardner J. 2015. UK science press officers, professional vision and the generation of expectations. *Public Understanding of Science.* Published Online First: 11 August 2015. doi: 10.1177/0963662515597188. 10.1177/0963662515597188 PMC5207296 26265709

[23] Alladi, S., T.H. Bak, V. Duggirala, B. Surampudi, M. Shailaja, A. Kumar Shukla, J.R. Chaudhuri, and S. Kaul. 2013. Bilingualism delays age at onset of dementia, independent of education and immigration status. *Neurology* 81(22): 1938–1944. 10.1212/01.wnl.0000436620.33155.a4 24198291

[24] Pickard S (2011). Health, illness and normality: The case of old age. BioSocieties.

[25] Partridge BJ, Bell SK, Lucke JC, Yeates S, Hall WD (2011). Smart drugs “as common as coffee”: Media hype about neuroenhancement. PLoS ONE.

[26] Racine E, Bar-Ilan O, Illes J (2006). Brain imaging A decade of coverage in the print media. Science Communication.

[27] Busby H, Williams G, Rogers A (1997). Bodies of knowledge: Lay and biomedical understandings of musculoskeletal disorders. Sociology of Health & Illness.

[28] Dumit J (2006). Illnesses you have to fight to get: Facts as forces in uncertain, emergent illnesses. Social Science & Medicine.

[29] O’Connor C, Joffe H (2015). How the public engages with brain optimization the media-mind relationship. Science, Technology & Human Values.

[30] Pickersgill M, Cunningham-Burley S, Martin P (2011). Constituting neurologic subjects: Neuroscience, subjectivity and the mundane significance of the brain. Subjectivity.

[31] Rose N (2007). The politics of life itself: Biomedicine, power, and subjectivity in the twenty-first century.

[32] Rubin BP (2009). Changing brains: The emergence of the field of adult neurogenesis. BioSocieties.

[33] Petersen A (2001). Biofantasies: Genetics and medicine in the print news media. Social Science & Medicine.

[34] Wynne B (1992). Misunderstood misunderstanding: Social identities and public uptake of science. Public Understanding of Science.

[35] Larsson SC, Wolk A (2012). Red and processed meat consumption and risk of pancreatic cancer: Meta-analysis of prospective studies. British Journal of Cancer.

[36] Monaghan L (1999). Challenging medicine? Bodybuilding, drugs and risk. Sociology of Health & Illness.

[37] Pickersgill M, Martin P, Cunningham-Burley S (2015). The changing brain: Neuroscience and the enduring import of everyday experience. Public Understanding of Science.

[38] Ingalhalikar, M., A. Smith, D. Parker, T.D. Satterthwaite, M.A. Elliott, K. Ruparel, H. Hakonarsond, R.E. Gurb, R.C. Gurb, and R. Vermaa. 2014. Sex differences in the structural connectome of the human brain. *Proceedings of the National Academy of Sciences* 111: 823–828. 10.1073/pnas.1316909110 PMC3896179 24297904

